# The dominant nature of Herzberg–Teller terms in the photophysical description of naphthalene compared to anthracene and tetracene

**DOI:** 10.1038/s41598-022-24081-0

**Published:** 2022-12-12

**Authors:** Anjay Manian, Salvy P. Russo

**Affiliations:** ARC Centre of Excellence in Exciton Science, School of Science, RMIT University, Melbourne, 3000 Australia

**Keywords:** Chemical physics, Excited states, Computational chemistry, Density functional theory, Method development, Quantum chemistry, Reaction mechanisms

## Abstract

The first order and second order corrected photoluminescence quantum yields are computed and compared to experiment for naphthalene in this manuscript discussing negative results. Results for anthracene and tetracene are recalled from previous work (Manian et al. in J Chem Phys 155:054108, 2021), and the results for all three polyacenes are juxtaposed to each other. While at the Franck–Condon point, each of the three noted polyacenes were found to possess a quantum yield near unity. Following the consideration of Herzberg–Teller effects, quantum yields stabilised for anthracene and tetracene to 0.19 and 0.08, respectively. Conversely, the second order corrected quantum yield for naphthalene was found to be 0.91. Analysis of this result showed that while the predicted non-radiative pathways correlate well with what should be expected, the approximation used to calculate second order corrected fluorescence, which yielded very positive results for many other molecular systems, here is unable to account for strong second order contributions, resulting in a grossly overestimated rate of fluorescence. However, substitution of an experimental radiative rate results in a quantum yield of 0.33. This work extols the importance of Herzberg–Teller terms in photophysical descriptions of chromophores, and highlights those cases in which a treatment beyond the above approximation is required.

## Introduction

The ability to wield a model which can predict the photophysical properties of any given chromophore is becoming more and more important in the field of photon harvesting^1–3^. Of particular difficulty is accounting for non-trivial couplings between states beyond the Franck–Condon level^4–6^ and include second order contributions. In the case where a given transition is very intense, such as a large transition dipole moment in the case of fluorescence, staying within the Franck–Condon approximation is often enough. However, in such a case whereby the transition is weak, such as a small transition dipole moment in the case of fluorescence, one must push beyond the Franck–Condon regime into the Herzberg–Teller regime^7^, where the matrix element is expanded as a Taylor series and we can include more terms^8–11^.

In simple terms, the expansion of the matrix element is truncated to exclude the nuclear dependence of the molecular system within the Franck–Condon regime, while it is included within the Herzberg–Teller regime. This often leads to the analogy of “intensity borrowing”, whereby vibrational normal mode distortions borrow some intensity of the matrix element from neighbouring states, in the case of fluorescence it is intensity borrowing from neighbouring states of the transition dipole matrix elements. While this means that a given Herzberg–Teller contribution is never zero, its importance is highlighted for transitions in which the wavefunctions components are heavily mixed, or when the first order term is smaller or comparable in size to the second order term.

In previous works^10^, we highlighted the importance of Herzberg–Teller terms to fluorescence, internal conversion (IC), and inter-system crossing (ISC) in the description of many chromophores. Importantly, while in this work the Herzberg–Teller components to IC and ISC were calculated explicitly, we used an approximation to estimate the radiative rate constant. Despite this, the use of this methodology yielded rate constants and consequential photoluminescence quantum yields (PLQYs) which compared very well to experimental values for both anthracene and tetracene, as well as pentacene, diketo-pyrrolopyrrole and perylene diimide. This same method has also been used to determine the exciton dynamics in many other studies^11,12^ and yield very positive results. However, in testing molecular systems for the initial work as per Ref.^10^, we noted that the employed approximation could not accurately model systems in which Herzberg-Terms dominate over Franck–Condon terms, and we feel it may be worth showing why.

**Figure 1:**
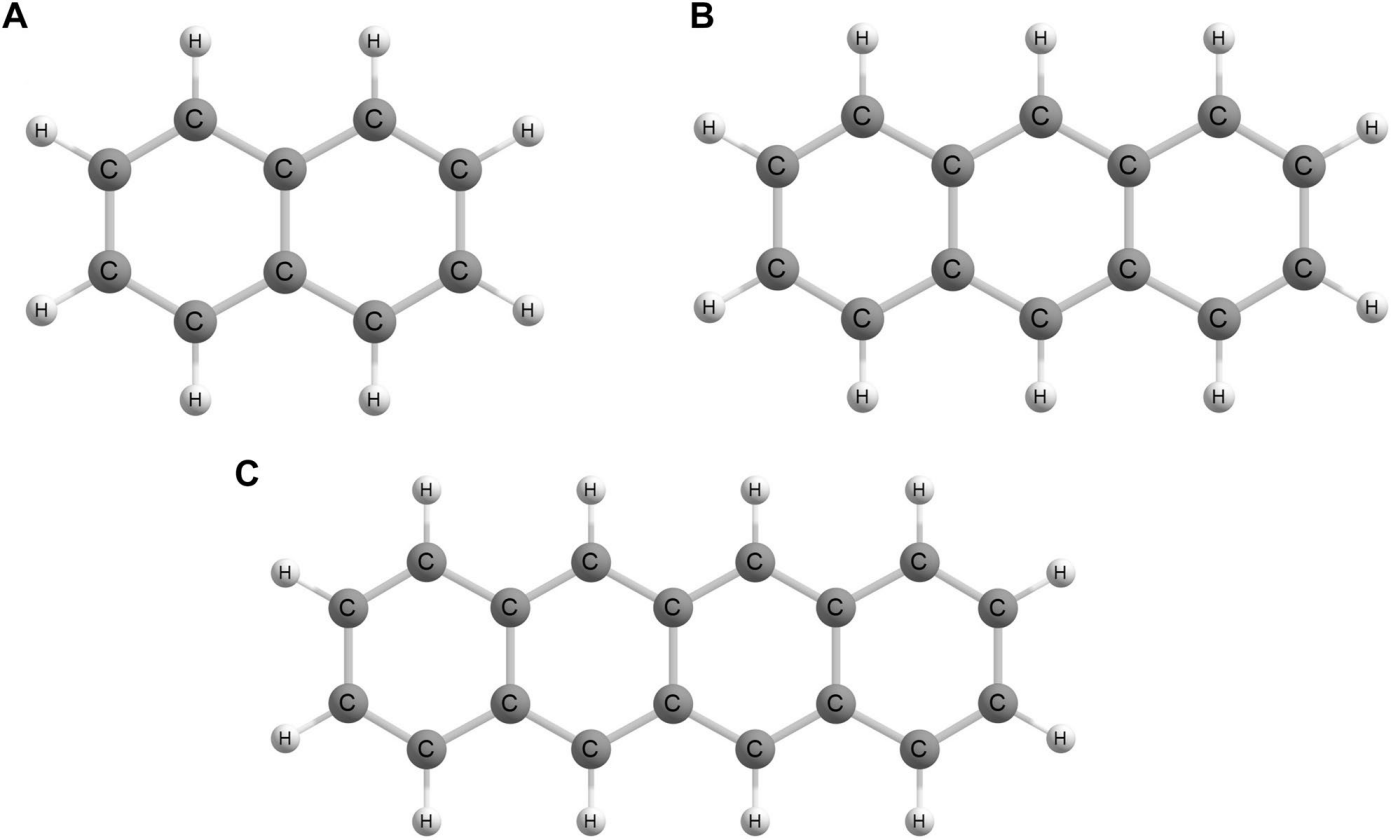
Schematic representation of (**A**) naphthalene, (**B**) anthracene, and (**C**) tetracene.

This report details one of the only failures of our current model (the other is to do with the triplet instability issue in TDDFT, however this can easily be solved by using different computational methods): the case where second order terms are vital in the description of fluorescence. Herein, we recall the results from Ref.^10^ for anthracene and tetracene, and use the same methodology to compute the PLQY of naphthalene, each of which is shown in Fig. 1. We organise our work as follows: “Theory” summarises the methodology used to calculate each respective rate constant, before summarising the methods used to calculate the quantum chemistry using density functional theory (DFT) based methods and the approximation used to compute the rate of fluorescence in “Methodology”. A complete treatment of the theory and methodology used in this work can be found in Refs.^10–13^, as this work is primarily focused on second order corrected fluorescence. The various rate constants important in describing the PLQY are then calculated and compared to experiment in “Results”. The cause of the discrepancy is then explored, before we then discuss what needs to be done to overcome this issue, in addition to presenting working alternatives currently within the state of the art in “Conclusion”.

## Theory

The rate of radiative decay $k_r$ is easily calculated as per Einstein’s spontaneous emission function^9–12,14^, given as:

1 $$\begin{aligned} k_r = \dfrac{1}{\tau _r} = \dfrac{4}{3\hbar c^3}\left<\Psi _0\left| \hat{d}\right| \Psi _1\right>^2\int S_d(\omega )\omega ^3d\omega \end{aligned}$$

Here, $\tau _r$ is the fluorescent lifetime, $\hbar$ is Planck’s reduced constant, *c* is the speed of light, $S_d$ is the normalised emission bandshape with energy $\omega$, and $\left<\Psi _0\left| \hat{d}\right| \Psi _1\right>$ is the transition dipole moment, also denoted as $\mu$. Einstein’s spontaneous emission function is valid when both initial and final states are in thermal equilibrium. This condition is upheld in this work.

To go beyond the Franck–Condon regime, we need to consider how the matrix element changes with respect to changes in the nuclear geometry^9^. To do this, the transition dipole can be expanded as a power series with respect to the vibrational coordinates $Q_j$ of the equilibrium geometry $Q_0$. Truncation of the Taylor expansion after the first-derivative term leads to the following:

2 $$\begin{aligned} \mu _\gamma =\mu _{0,\gamma }+\sum _j\left[ \dfrac{\partial \mu _\gamma }{\partial Q_j}\right] _{Q_0}Q_j, \quad \gamma =x,y,z \end{aligned}$$

Here, the right hand side can be divided into two segments. The first term mediates the Franck–Condon component, while the second term mediates the Herzberg–Teller component, or the change to the matrix element due a perturbation along the *j*th vibrational normal mode.

The quantum yield is a measure of efficiency for a particular pathway for a particular electronic excited state. In the case of the PLQY, it is a measure of the probability a photon will be emitted upon photo-excitation. For a monomer, the only competing pathways are the rates for IC $k_{ic}$ and ISC $k_{ISC}$ against the rate of fluorescence $k_r$:

3 $$\begin{aligned} \text {PLQY} = \frac{k_{r}}{k_r + k_{ic} + k_{ISC}} \end{aligned}$$

## Methodology

Photophysical properties and rate constants for anthracene and tetracene were obtained from Ref.^10^. Calculations for naphthalene were obtained using the same methods. Molecular geometries and their corresponding electronic Hessians were optimised using the Becke 3-parameter Lee–Yang–Parr B3LYP exchange-correlation hybrid functional^15–18^ using the redefined valence triple-zeta polarization basis set def2-TZVP^19^ basis set using the Gaussian16 software package^20^. Solvation effects were simulated via the employment of a polarizable continuum model (PCM). Single-point energies, transition dipole moments, and spin-orbit couplings were all calculated using DFT based multireference configuration interaction DFT/MRCI method^21–28^, using the same basis set. We recommend reviewing Refs.^10–13^ for further details where necessary.

The VIBES software package^29^ was used to generate the Franck–Condon and Herzberg–Teller spectral densities, as well as calculate ISC rates, using $2^{16}$ integration points with a 300 fs time integral for integration, and a 100 cm$^{-1}$ width for the Gaussian damping of the calculated correlation function. A temperature of 300 K was assumed for all calculations. For IC rates, the Knapsack software package^30^ was used to compute IC rate constants. Employed parameters include using a Franck–Condon weighted density, and a contributions threshold of 15. Derivative components were calculated using a central difference method.

When computing the Herzberg–Teller emission rate, care must be taken as the spectral intensity is often difficult to extract. At the Franck–Condon point, this intensity is to the first order, and scales proportional to the transition dipole moment. However, the Herzberg–Teller treatment mediates an inclusion of the second order term due to nuclear activity of normal modes. As this calculation is expensive, available codes compute the spectra in the time domain, which means that some information, such as the second order corrected transition dipole moment, is not readily available, and is baked into the spectral density. However, we found that an *effective* transition dipole moment can be extracted from the density if we compare the first order bandshape to the second order one using the first order normalisation factor. In other words, while not the exact second order corrected transition dipole moment, the employed transition dipole moment is accurate assuming certain conditions are met.

Using Vibes, the first order spectra can be calculated either dependant or independent of the transition dipole matrix elements. Both resulting spectra differ in spectral intensity, with this difference being related directly to the square of the transition dipole moment. From the normalisation factors of both dipole-dependant ($N_D$) and dipole-independent ($N_I$) spectra, this connection becomes more clear:

4 $$\begin{aligned} \mu =\sqrt{\frac{N_D}{N_I}} \end{aligned}$$

From this link, we can approximate the second order corrected transition dipole moment. Vibes does not currently decouple the second order corrected transition dipole moment from the dipole-dependant spectra; as such we do not have access to the normalisation factor. However, if we assume minimal difference between the Franck–Condon and Herzberg–Teller corrected spectra, such that the second order contribution is not dominant, we can approximate an *effective* transition dipole moment which we can use to compute the rate constant.

5 $$\begin{aligned} k_{r,\gamma ,FC+HT} = \dfrac{4\mu _\gamma ^2}{3\hbar c^3 N^2_I}\int S_{d,FC+HT}(\omega )\omega ^3d\omega \end{aligned}$$

This approximation can be expected to work in any case where dominant contributions to the transition dipole moment come from Franck–Condon terms. In other words, where the change to the *effective* transition dipole is much smaller than or comparable in size/magnitude to the transition dipole at the Franck–Condon point:

6 $$\begin{aligned} A_{FC} \approx A_{FC+HT} \quad \Rightarrow \quad \mu _\gamma \approx \mu _{0,\gamma } \end{aligned}$$

Naphthalene is known to depend on Herzberg–Teller components to fluoresce^31–37^ and as such, provides a perfect case study to examine the validity of this approximation. If the condition shown in Eq. (6) is not met, we can expect the spectrum to be un-normalised, therefore resulting in an overestimated fluorescence rate constant.

## Results

### Quantum chemistry

**Figure 2:**
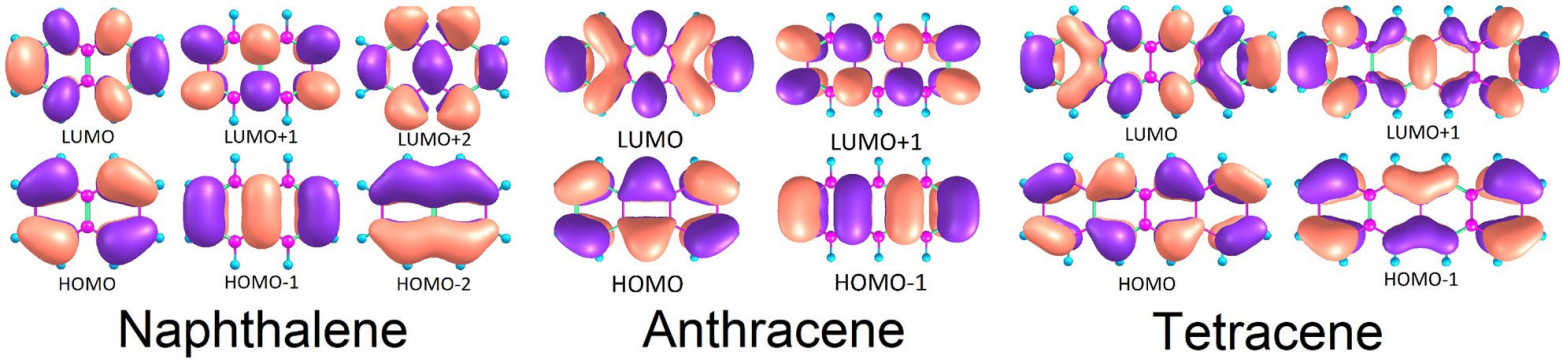
Highest occupied (lowest unoccupied) molecular orbitals HOMO (LUMO) for naphthalene, anthracene, and tetracene.

Analysis of the quantum chemical results shows the highest occupied molecular orbitals (HOMO) and lowest unoccupied molecular orbitals (LUMO) comprising the wavefunctions of naphthalene at the ground state geometry to be mixed for the $S_1$, state of HOMO-1$\rightarrow$LUMO and HOMO$\rightarrow$LUMO+1 type orbitals (Fig. 2). Meanwhile, the HOMO$\rightarrow$LUMO transition is found on the $S_2$ state. Similarly mixed wavefunction terms can be observed for the first singlet excited state geometry, with an $S_1$ wavefunction built of HOMO$\rightarrow$LUMO+1 and HOMO-1$\rightarrow$LUMO terms, suggesting naphthalene is likely to emit from it’s longitudinal $L_b$ excited state, as per Platt’s notation^38^. Comparison to work by Hashimoto et al.^39^ show very similar results, as shown in Table 1, with a vertical excitation of 4.09 eV at the $S_1$ geometry using a multireference Møller–Plesset method method, differing from our computed value by less than 0.20 eV.

**Table 1:** Singlet and triplet energies for naphthalene, anthracene, and tetracene, compared to those found in literature, in eV.

State	DFT/MRCI			Experiment			Theory		
	Abs.	Emi.	0-0	Abs.	Emi.	0-0	Abs.	Emi.	0-0
**Naphthalene**									
$S_1$	4.20	3.96	4.28	$4.02^{\text {a}}$	$3.98^{\text {b}}$, $3.94^{\text {c}}$	$3.97^{\text {d}}$	$4.09^{\text {e}}$	–	$4.13^{\text {f}}$
$S_2$	4.57	4.36	4.51	$4.49^{\text {g}}$	$4.34^{\text {b}}$	$4.45^{\text {f}}$	$4.62^{\text {e}}$	–	$4.42^{\text {f}}$
$T_1$	3.00	2.18	2.65	$2.98^{\text {h}}$	–	–	–	–	–
$T_2$	3.91	3.69	3.83	–	–	–	$3.42^{\text {i}}$	–	–
$T_3$	4.34	4.14	4.28	$3.87^{\text {h}}$	–	–	–	–	–
**Anthracene**									
$S_1$	$3.39^{\text {j}}$	$2.96^{\text {j}}$	$3.18^{\text {j}}$	$3.31^{\text {k}}$	$3.23^{\text {l}}$, $3.30^{\text {c}}$	$3.43^{\text {m}}$, $3.38^{\text {n}}$	$3.40^{\text {o}}$	$3.27^{\text {p}}$, $3.01^{\text {q}}$	$3.43^{\text {q}}$, $2.67^{\text {q}}$
$S_2$	$3.61^{\text {j}}$	$3.41^{\text {j}}$	$3.53^{\text {j}}$	$3.45^{\text {s}}$	–	–	$3.23^{\text {o}}$	$3.66^{\text {q}}$	–
$T_1$	$2.04^{\text {j}}$	$1.30^{\text {j}}$	$1.77^{\text {j}}$	$1.85^{\text {t}}$, $1.82^{\text {u}}$	$2.00^{\text {o}}$, $1.66^{\text {i}}$	–	–		
$T_2$	$3.41^{\text {j}}$	$2.92^{\text {j}}$	$3.19^{\text {j}}$	–	–	–	$2.84^{\text {i}}$, $3.30^{\text {o}}$	–	$2.40^{\text {v}}$
$T_3$	$3.42^{\text {j}}$	$3.37^{\text {j}}$	$3.58^{\text {j}}$	–	–	–	$3.50^{\text {i}}$, $3.35^{\text {o}}$	–	$3.77^{\text {v}}$
**Tetracene**									
$S_1$	$2.61^{\text {j}}$	$2.28^{\text {j}}$	$2.44^{\text {j}}$	$2.60^{\text {b}}$	$2.60^{\text {w}}$	$2.71^{\text {n}}$	$2.80^{\text {o}}$, $2.74^{\text {x}}$, $2.64^{\text {y}}$	$2.63^{\text {p}}$, $2.30^{\text {z}}$	–
$S_2$	$3.24^{\text {j}}$	$3.08^{\text {j}}$	$3.18^{\text {j}}$	$3.14^{\text {b}}$	–	–	$2.92^{\text {o}}$, $3.22^{\text {x}}$, $3.48^{\text {y}}$	–	–
$T_1$	$1.38^{\text {j}}$	$0.62^{\text {j}}$	$1.36^{\text {j}}$	–	–	$1.28^{\text {t}}$	$1.51^{\text {o}}$	–	$1.10^{\text {v}}$, $1.25^{\text {aa}}$
$T_2$	$2.63^{\text {j}}$	$2.25^{\text {j}}$	$2.25^{\text {j}}$	–	–	–	$2.43^{\text {o}}$, $1.87^{\text {i}}$	–	$2.58^{\text {v}}$

Given absorption (Abs.) and emission (Emi.) energies are vertical excitation energies from the ground state, while adiabatic energies are presented here as 0-0 transitions. A “–” indicates that the information could not be found.

$^{\text {a}}$From Ref.^40^ in the vapour phase. $^{\text {b}}$From Ref.^41^ using cyclohexane. $^{\text {c}}$From Ref.^42^ using cyclohexane. $^{\text {d}}$From Ref.^43^ in the vapour phase. $^{\text {e}}$From Ref.^39^ using MRMP/cc-pVDZ. $^{\text {f}}$From Ref.^44^ using CC2/TZVPP. $^{\text {g}}$From Ref.^45^ using n-hexone. $^{\text {h}}$From Ref.^46^ using CNDO/S-CI. $^{\text {i}}$From

Ref.^47^ using CI. $^{\text {j}}$From Ref.^10^ using DFT/MRCI. $^{\text {k}}$From Ref.^48^ using h-heptane. $^{\text {l}}$From Ref.^49^ using chlorobenzene $^{\text {m}}$From Ref.^50^ in the vapour phase. $^{\text {n}}$From Ref.^51^ in the gas phase. $^{\text {o}}$From Ref.^52^ using MRMP/cc-pVDZ. $^{\text {p}}$From

Ref.^53^ using SCFMO. $^{\text {q}}$From Ref.^54^ using SAC-CI(SD-R)/6-31G(d,p). $^{\text {r}}$From Ref.^55^ using PBE/TZP. $^{\text {s}}$From Ref.^56^ using cyclohexane. $^{\text {t}}$From Ref.^57^. $^{\text {u}}$From Ref.^58^ in ammonia. $^{\text {v}}$From Ref.^59^ in alcohol. $^{\text {w}}$From Ref.^60^ using toluene. $^{\text {x}}$From Ref.^61^ using DFT/MRCI/TZVP. $^{\text {y}}$From Ref.^62^ using B3LYP/ANO-S-VDZP. $^{\text {z}}$From Ref.^63^ using RAS-2SF/6-31G*. $^{\text {aa}}$From Ref.^64^ in the vapour phase.

**Table 2:** Tabulated singlet to triplet quantum chemical transition properties for naphthalene, and collated data for anthracene and tetracene from Ref.^10^, at the $S_1$ Franck–Condon point.

Compound	State	$\Delta E_{S_1\rightarrow T_n}$	$H_{SO}(x)$	$H_{SO}(y)$	$H_{SO}(z)$
Naphthalene	$T_1$	1.630	− 0.05269	0.00006	− 0.00009
	$T_2$	0.451	$-$ 0.00000	$-$ 0.00002	$-$ 0.00000
	$T_3$	$-$ 0.003	$-$ 0.00000	$-$ 0.00000	0.00009
Anthracene	$T_1$	1.412	$-$ 0.00006	0.00001	$-$ 0.00002
	$T_2$	$-$ 0.009	$-$ 0.00004	$-$ 0.00001	0.00000
	$T_3$	$-$ 0.396	$-$ 0.00036	$-$ 0.00015	0.00527
Tetracene	$T_1$	1.073	0.00000	0.00003	$-$ 0.00001
	$T_2$	$-$ 0.014	0.00001	$-$ 0.00005	$-$ 0.00002

Compound	$\mu _0$	$\mu$			
Naphthalene	0.042	1.734			
Anthracene	1.313	1.404			
Tetracene	1.340	1.443			

Energy differences are adiabatic, with negative energies defined as transition to a higher state. Energies are given in units of eV, while SOCME terms are in units of cm$^{-1}$. Sub-table shows the Franck–Condon transition dipole moment $\mu _0$ and *effective* transition dipole moment $\mu$ calculated as per Eq. (4), in atomic units.

In the triplet manifold, steady state characteristics can be observed across all geometries, with the first and second triplet excited states manifesting as a transverse HOMO$\rightarrow$LUMO transition and longitudinal HOMO-1$\rightarrow$LUMO and HOMO$\rightarrow$LUMO+1 mixed character. We also note a near-degeneracy between the $S_1$ state and $T_3$ state, likely facilitating a fast ISC mechanism. Spin-orbit coupling is only strong for the lowest triplet excited state with a moment of 0.05 cm$^{-1}$ and the rest negligible, as per Table 2. This suggests that second order contributions to ISC rate constants are important. An error in the computed adiabatic energy of the $S_1$ state should be highlighted, whereby the adiabatic energy is higher than the absorption energy. However, this can be attributed to the differences in computational methods, where geometries were computed using DFT and singlet-point energies were computed using DFT/MRCI. Considering that the emitting state is longitudinal and very close in energy to the transverse manifold, this system would be dominated by second order radiative processes.

Unlike naphthalene, anthracene displays very little mixing of configuration state functions. $S_1$ wavefunctions across all optimised geometries show a strong and clear HOMO$\rightarrow$LUMO type contribution, with an adiabatic energy of 3.18 eV and a transition dipole moment of 1.31 au at the $S_1$ Franck–Condon point, below the dark $L_b$ state. This is similar in tetracene, with a clear bright $L_a$ state adiabatically positioned at 2.44 eV with a Franck–Condon transition dipole moment of 1.34 au, followed by a dark $L_b$ state. Looking at the triplet manifold in both 3 and 4 ringed polyacenes, we note strong overlaps for the $S_1\rightarrow T_2$ transitions seen here as very small energy gaps between initial and final states. In particular, anthracene displays a near-resonance of the two states, similar to the naphthalene for the $S_1\rightarrow T_3$ transition. Spin-orbit coupling between the $L_a$ state and the triplet levels are non-zero in all cases, but sizable for only the $T_3$ states, highlighting the need for nuclear contributions to the spin-orbit coupling matrix element.

**Figure 3:**
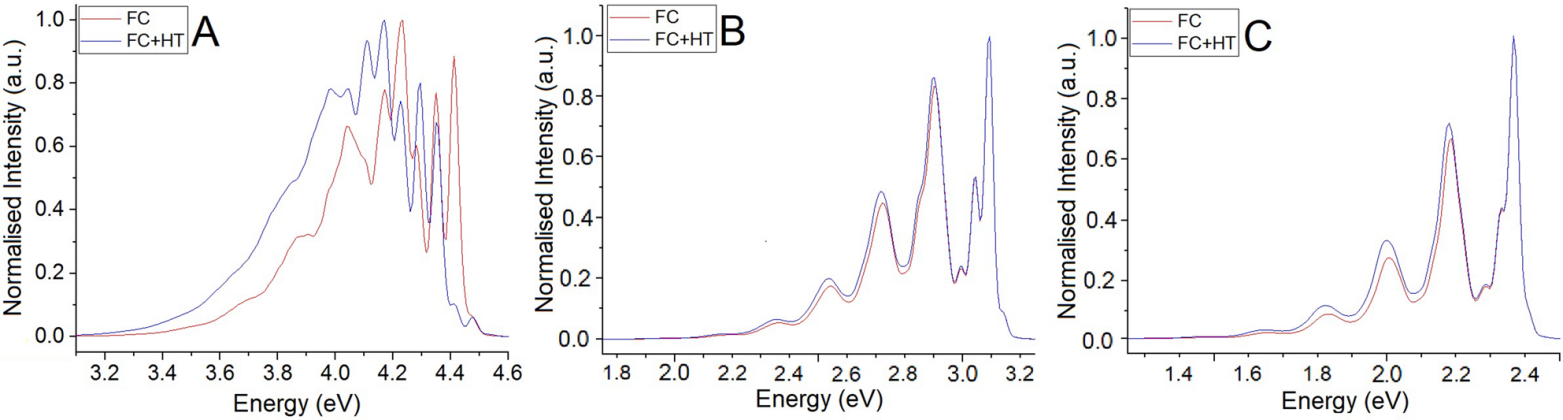
Calculated emission spectra of (**A**) naphthalene, (**B**) anthracene, and (**C**) tetracene. Solvation in cyclohexane was simulated via a PCM at both the Franck–Condon (Red) and Herzberg–Teller (Blue) levels. Spectra are normalised by intensity, and as such are not to scale. (**B**,**C**) were taken from Ref.^10^ with permissions.

When we compare these results with those in the current literature^50,52,57^, we see that for the most part they correlate well. It should be noted that in previous work^10^, we found evidence that the triplet geometries computed using time dependent DFT were overestimated. This is seen clearly in comparing the adiabatic energies computed in Ref.^10^ with literature values in Table 1. It is likely this same overestimation is present in the naphthalene chromophore as it was in anthracene and tetracene. As such, assuming the singlet properties are reasonable, which we have no reason to suspect otherwise, then we can expect some degree of overestimation from ISC pathways.

From the quantum chemistry alone, for naphthalene we can expect Herzberg–Teller effects to play a major role in the description of all the photophysics of the chromophore. In the case of fluorescence, the $L_b$ state is optically dark but the emitting state. In other words, the condition noted in Eq. (6) is not maintained. As such, nuclear effects shifting the photochemical properties closer to the $L_a$ state would have a drastic effect due to the dark nature of the $L_b$ state. In the case of IC and ISC, both the large energy gaps and the weak coupling between states highlights that Franck–Condon processes may not be able to describe the behaviour of the chromophore alone. For the other polyacenes, this is similarly seen. IC and ISC processes look similarly weak at a first order approximation, and can be expected to be small without a second order correction. However, in the case of fluorescence Eq. (6) for these two chromophores holds true. Both anthracene and tetracene display strong transition dipole moments. Therefore, Herzberg–Teller terms can be expected to *not* play a dominating role in radiative decay, and as can be expected to yield appreciable results from our model.

### Fluorescence

Naphthalene was observed to possess a very small number of non-zero Franck–Condon displacements due to its high degree of molecular symmetry, and as such displays a very distinct vibronic progression. As shown in Fig. 3A, we see a very large difference in the leading peak in the second order corrected spectra. It also displays a significantly larger *effective* transition dipole moment of 1.734 au as computed using our approximation, which is significantly larger than the moment at the Franck–Condon point, computed here as 0.042 au. Comparison to experimental results^42,50,65^ shows significantly less definition of the bandstructure, with more broadening across peaks.

It is also worth noting that the higher energy segment of the spectra is much lower in intensity than we predicted, possibly due to some solute-solvent coupling effects. However, naphthalene is non-polar, and therefore is not known to display strong solvatochromic properties^66^, where the main impact of solvent is the degree of solubility. While known to be solvent sensitive in an excimeric state^67^, this is not the case here. As such, the choice of solvent should not affect the photophysical properties to a large degree.

Anthracene, shown in Fig. 3B, was observed with 4 distinct peaks across both Franck–Condon and Herzberg–Teller regimes. Shown in Fig. 3C, this is a similar case again for tetracene, which displays a steady progression of three distinct peaks across both first and second order regimes. Both compare well to literature^60,68^, and we can see that at least in the case of fluorescence, the differences between first and second order corrected spectra start to minimise, with some intensity increase on the secondary and tertiary peaks.

Computed rate constants are given for all important transitions are given in Table 3. The low rate of $1.35\times 10^5$ s$^{-1}$ for naphthalene can easily be attributed to emission from the optically dark $L_b$ state. Here, complex behaviour and strong wavefunction mixing of the low-lying excited states yield a poor transition dipole moment, and therefore a small rate of fluorescence. This is similarly noted by Nijegorodov et al.^41^. With the previously noted low $S_1$ transition dipole moment, staying within the Franck–Condon regime fails to account for the total radiative rate by several orders of magnitude, as previously predicted. Inclusion of Herzberg–Teller terms to the rate constant using our approximation yields a result too far in the other direction, yielding a rate constant and fluorescent lifetime of $2.11\times 10^8$ s$^{-1}$ and 8 ns, respectively. Here, we expect a lifetime of 96 ns as published by Nijergorodov et al.^41^, corresponding to a rate of approximately $1.04\times 10^7$ s$^{-1}$, which is of course more than an order of magnitude slower.

**Table 3:** Tabulated rate constants with the Franck–Condon (FC) and Herzberg–Teller corrected (FC+HT) regimes for naphthalene, anthracene, and tetracene, given in units of s$^{-1}$.

Transition	FC (1st order) rate	FC+HT (2nd order corrected) rate
**Naphthalene**		
Fluorescence	$1.35\times 10^{5}$	$2.11\times 10^{8}$
$S_1\rightarrow S_0$	$5.58\times 10^{-21}$	$3.91\times 10^{0}$
$S_1\rightarrow T_1$	$3.17\times 10^{-3}$	$8.34\times 10^{5}$
$S_1\rightarrow T_2$	$4.47\times 10^{-2}$	$8.10\times 10^6$
$S_1\rightarrow T_3$	$1.66\times 10^{0}$	$1.19\times 10^7$
**Anthracene**		
Fluorescence	$4.28\times 10^{7}$	$4.83\times 10^{7}$
$S_1\rightarrow S_0$	$2.72\times 10^{2}$	$3.69\times 10^{6}$
$S_1\rightarrow T_1$	$2.79\times 10^{-9}$	$3.90\times 10^{0}$
$S_1\rightarrow T_2$	$1.51\times 10^{-1}$	$2.08\times 10^8$
$S_1\rightarrow T_3$	$3.66\times 10^{-3}$	$7.96\times 10^1$
**Tetracene**		
Fluorescence	$2.01\times 10^{7}$	$2.26\times 10^{7}$
$S_1\rightarrow S_0$	$1.74\times 10^{5}$	$2.60\times 10^{7}$
$S_1\rightarrow T_1$	$7.25\times 10^{-9}$	$1.37\times 10^{-1}$
$S_1\rightarrow T_2$	$3.61\times 10^{-1}$	$2.37\times 10^8$

Rates for anthracene and naphthalene were taken with permission from Ref.^10^.

Anthracene was found to yield a first order rate constant of $4.28\times 10^7$ s$^{-1}$ which corresponds to a fluorescence lifetime of 23 ns. Approximately 7 ns too fast as per work by Nijegorodov et al.^41^, a second order correction yields a new transition dipole moment of 1.40 au, resulting in a rate and lifetime of $4.83\times 10^7$ s$^{-1}$ and 21 ns, respectively. A similar improvement is seen for tetracene with a first order rate and lifetime of $2.01\times 10^7$ s$^{-1}$ and 50 ns, respectively, improved to $2.26\times 10^7$ s$^{-1}$ and 44 ns upon a second order correction.

In trying to understand why the approximation fails, we can examine the dependant variables of Eq. (5), which infers the error is centred upon either the method we compute the *effective* transition dipole moment or excitation energies. Comparison of our computed photophysical properties to literature shows that our energies and oscillator strengths compare well to literature. Absorption and emission energies are well within acceptable margins, while the overestimated adiabatic energy has already been attributed to a change in computational methods. While our computed transition dipole moment of 0.04 au for naphthalene (Table 2) is overestimated by an order of magnitude with respect to experiment as published by George and Morris^45^, it does compare well to the MRMP computed value by Hashimoto et al.^39^. This therefore suggests that the approximated second order normalisation factor is being underestimated. Examination of the cases where this approximation holds^10–12^ shows very clearly that Eq. (6) must hold; the approximation fails when Franck–Condon components are not dominant. In this work for naphthalene, second order terms are clearly dominant in the photophysical description of the chromophore, shown by the underestimated first order fluorescence rate constant. In the case where the second order correction holds the dominating terms, the approximation fails, shown by the overestimated second order corrected fluorescence rate constant. In order to correctly account for the actual Herzberg–Teller contribution, a full and explicit treatment will have to be developed.

### Non-radiative decay

Non-radiative rate constants for each polyacene are tabulated in Table 3. At the Franck–Condon limit, the IC rate constant for naphthalene is very small with rates of $5.58\times 10^{-21}$ s$^{-1}$, and a second order corrected rate constant of 3.91 s$^{-1}$. Nijegorodov et al.^41^ found an IC rate constant of $2.0\times 10^{4}$ s$^{-1}$, which is four orders of magnitude larger than our value. Comparing this rate with work by Valiev et al.^69^ who factored in anharmonic effects, or to Kohn et al.^70^ who noted complexities associated with near-equilibrium energetics, shows that the harmonic approximation for modelling IC in naphthalene is not enough to account for the complex photophysics. However, even the cited rates are too slow to compete with fluorescence, and as such can be considered negligible. For anthracene and tetracene, IC rates were similarly small within the Franck–Condon regime, with rates of $2.72\times 10^2$ s$^{-1}$ and $1.74\times 10^5$ s$^{-1}$, respectively. Upon factoring in Herzberg–Teller effects, these rates increase drastically to $3.69\times 10^6$ s$^{-1}$ and $2.60\times 10^7$ s$^{-1}$, respectively, agreeing well with results reported by Pedash et al.^71^.

When considering how small first order spin-orbit coupling matrix elements are for all possible transitions from the emitting states for each of the polyacenes, very small rate constants should be expected. For naphthalene, the combined ISC rate constant was found to be 1.66 s$^{-1}$. Similarly small, anthracene and tetracene both yielded combined first order rate constants of $1.51\times 10^{-1}$ s$^{-1}$ and $3.61\times 10^{-1}$ s$^{-1}$, respectively. It should be noted that the dominating contribution to each polyacene’s combined ISC rate constant came from each chromophore’s near-resonant triplet level to the emitting state. Combined second order corrected ISC rates for naphthalene, anthracene, and tetracene were found to be $2.09\times 10^7$ s$^{-1}$, $2.08\times 10^8$ s$^{-1}$, and $2.37\times 10^8$ s$^{-1}$, respectively. While these values are faster than those reported by Nijegorodov et al.^41^, they do agree well with those published by Pedash et al.^71^ who did consider higher order processes, of $1.90\times 10^8$ s$^{-1}$, and $2.50\times 10^8$ s$^{-1}$ for anthracene and tetracene respectively, while work by Parker and Joyce^72^ agrees with the fast ISC speeds in naphthalene.

Here we highlight the importance of second order processes, which show quite conclusively that accurate photophysical descriptions require some correction for nuclear behaviour. As per Table 2, both anthracene and tetracene display very weak spin-orbit Hamiltonian matrix elements, especially when compared to systems with larger atoms^73–75^. Consequentially, we would normally expect small ISC rates. However this is not the case; the rates are still very fast despite the very small spin-orbit terms. Considering the perturbative interpretation of this problem, with respect to the energy gap law^76^, smaller energies as per the energy gap law will result in much faster transitions. However, the important consideration here is the Herzberg–Teller components. This is shown clearly when comparing the first and second-order corrected rates. This is because they are dominant, while Franck–Condon terms are not.

### Photoluminescence quantum yield

From the calculated rate constants, the quantum yields within the Franck–Condon regime for each of the studied polyacenes are all values of near-unity, with PLQYs of 1.00, 1.00, 0.99 for naphthalene, anthracene, and tetracene, respectively. These high efficiencies are due entirely to the omission of higher order photophysical properties found in compounds with complex mixing of excited state wavefunction components.

When we begin to consider second order corrections, we can instead yield PLQYs of 0.91, 0.19, and 0.08 for naphthalene, anthracene and tetracene, respectively. While anthracene and tetracene compare well with the experimental values of 0.24 and 0.21 as reported by Nijegorodov et al.^41^, the PLQY of naphthalene, reported with an experimental PLQY of 0.23, is grossly overestimated due to the failure of our radiative approximation. This becomes clear if we consider the experimental rate of $1.04\times 10^7$ s$^{-1}$ reported by Nijegorodov et al.^41^; if we instead use all of our calculated non-radiative rates, but use this experimental radiative rate to calculate a PLQY, we instead obtain an efficiency of 0.33, which is much more accurate than previously. Conversely, both anthracene and tetracene are in much better agreement with experiment^41^. Both are slightly underestimated, but this is likely caused by the overestimated triplet energies, and therefore overestimated ISC rate constants.

It should be highlighted that the mis-estimated energy levels could result in a misleading description for dominant Franck–Condon and Herzberg–Teller components to the radiative and non-radiative pathways. However, triplet instability is a curse of the DFT method itself, and can only be solved by employing a higher level method. Further, high level methods incorporating solute-solvent interactions are rare. While for naphthalene this is unimportant, for other Herzberg–Teller dominant chromophores, this may be an important consideration.

### The failure of the *effective* transition dipole moment model

As already discussed in “Methodology”, naphthalene is well known to experience strong Herzberg–Teller contributions to many of it’s transitions. This work in particular shows that from the first singlet excited state, the chromophore displays dominant second order contributions for all relaxation pathways, radiative and non-radiative. In the case of non-radiative transitions, matrix elements for anti-parallel electron-spin and parallel electron-spin transitions are weakly coupled in the Franck–Condon regime, however increase in strength drastically upon the inclusion of second order terms. The methodology to treat the non-radiative methods^10,29,30^ is already established and compares well with experiment. In the case of radiative transitions however, the story is different. As shown in previous works^10–12^, Eq. (5) is viable assuming the condition shown in Eq. (6) holds. However, as clearly shown in “Fluorescence”, this is not the case for naphthalene, as Herzberg–Teller terms are clearly dominant in it’s photophysical description.

Examination of the current state of the literature shows little in the way of alternate methods which are also exact. The most common method reported to treat this problem is using the Fcclasses code package developed by Santoro et al.^77–79^, which truncates the problem down to only the strongly contributing normal modes. This methodology was used by Dong et al.^80^, Guo et al.^81^, and Wykes et al.^8^; in all cases providing a significant degree of clarity to the results. For reported treatments straying from the Fcclasses methodology; Kunda et al.^82^ developed an exact treatment using a path integral formalism. Despite achieving positive results, they were limited to a small number of discrete normal modes. De Souza et al.^83^ also implemented a path integral treatment using the Orca software package^84^, and while it was not as “exact” as the work by Kunda et al., the results produced were very positive. Yin et al.^85^ used a similar derivatives method used in the Vibes software package in the MOlecular MAterials Property Prediction (Momap) software package^86–89^, showing high efficiency within the Herzberg–Teller regime for the otherwise emissively dark 5,10-dihydrophenazine.

With respect to what is available as an alternative method in the current state of the art, the Fcclasses method seems to be the most prominent and successful due to its flexibility, however its exclusion of most normal modes from the expansion of the transition dipole can be interpreted at best as an approximate rate constant. That is not to say our method is better; rather to the contrary. We argue this, as we found definitively that removal of even weakly coupled normal modes to a transition can still have a strong effect on the configuration space, and therefore on the Franck–Condon factors^10^. However, while one of those weakly contributing modes may have little effect on the rate constant, many of them together may have a rather important impact. And in a field like the development and modelling of photon harvesting applications, that small difference may be important.

**Figure 4:**
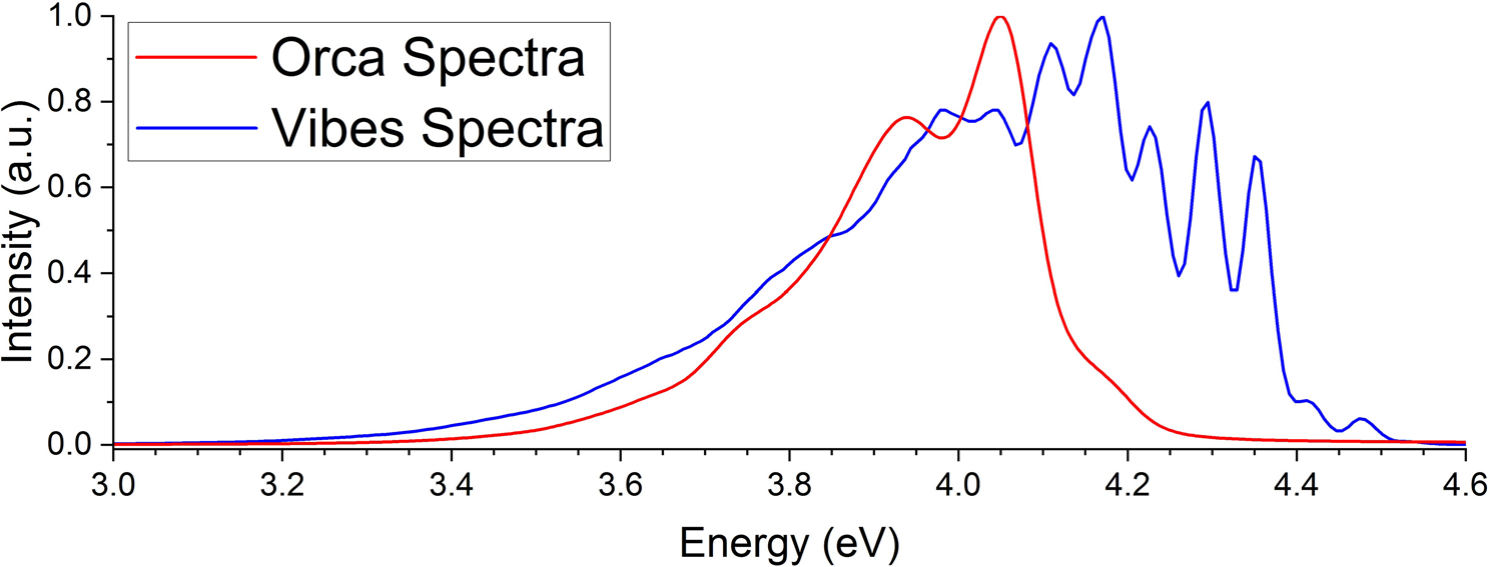
Calculated second-order corrected emission spectra using Orca (red). Reference second-order corrected emission spectra taken from Fig. 3A (blue). Spectra are normalised by intensity, and as such are not to scale.

If we employ the path integral treatment by de Souze and co-workers using Orca 5.0.3 mentioned previously^83,84^, we yield a second-order corrected fluorescence rate constant of $6.757\times 10^6$ s$^{-1}$, with 95.35% of that rate due to Herzberg–Teller terms. There are a number of important considerations that need to be addressed with this method. Firstly, there does not appear to be any implemented method to track energy level inversion, which is an important phenomenon for Naphthalene, and can lead to erroneous results. Further, the spectral shape is vastly different to the second-order corrected spectra predicted by Vibes as per Fig. 4, with very little vibronic definition in the high energy region where it would normally be expected. Finally, like Vibes, Orca calculates all components in the frequency domain, resulting in a hidden second-order corrected transition dipole moment and spectral normalisation factor. This means that outside the Orca platform, this data cannot be used easily. Despite these issues however, the corresponding PLQY is 0.245, which is much closer to experiment. It is difficult to speculate to the accuracy of this rate due to the drastically different lineshape with respect to spectra calculated using Vibes and Orca, possibly due to the approximations made to treat the problem. What is clear is that despite the innacurate spectra, within the Orca suite Herzberg–Teller terms are treated correctly.

As it stands, we could find no single package which yields an exact result free from truncation of the vibronic progression. And unfortunately, it will take some time before our methodology is updated beyond the current approximative method to account for second order effects exactly. We believe that a superior treatment would require the explicit calculation of the derivative components within the frequency domain, allowing the second order corrected transition dipole moment to be easily extracted from the calculation results, and this is a subject of ongoing work.

## Conclusion

From first principles, we have calculated the photophysical properties of naphthalene simulated in a solution of cyclohexane, and compared them with results for anthracene and tetracene calculated using the same methods. While quantum chemical results correlating well with literature, predicted rate constants using our approximation method for naphthalene however yielded a second order correction to fluorescence which was overestimated by an order of magnitude. This same error was not observed in either anthracene or tetracene, which were shown to be accurate with respect to the level of theory used to calculate the equilibrium geometries.

IC was noted to be negligible in it’s contribution to fluorescent quenching, with almost all the non-radiative intensity manifesting as a result of a near resonance with the emitting state and the triplet manifold; the $T_3$ state in the case of naphthalene, and the $T_2$ state in the case of both anthracene and tetracece. From these rate constants a first order and second order corrected PLQYs were calculated for naphthalene as 1.00 and 0.91, respectively. However, use of an experimental fluorescent rate yielded a new PLQY of 0.33, highlighting the error in our approximation method. For anthracene and tetracene, both displayed a first order PLQY near unity, which drastically stabilised down to 0.19 and 0.08 respectively upon consideration of Herzberg–Teller components. Alternatives to the *effective* dipole treatment were then explored, however, we could find no single methodology which could calculate a full, un-truncated spectral density to the second order exactly.

In conclusion, the model used, while robust, needs to be expanded upon to factor in an explicit treatment of second order fluorescence, such that systems sensitive to Herzberg–Teller effects can be examined. This work shows that our approximation for second order normalisation could accurately estimate the full transition dipole moment for chromophores where Herzberg–Teller processes are not dominant. Of note, second order contributions were important not only for fluorescence, but also for ISC and IC. However in the case of radiative decay, when the Franck–Condon component is very small with respect to the Herzberg–Teller component, such as when the change to the *effective* transition dipole is *not* smaller than or comparable to the transition dipole at the Franck–Condon point, the approximation fails and results in an overestimation of the transition. Despite this, our model still has a future in the role of property prediction, as the radiative approximation remains separate from the theory used to compute IC and ISC. But it is clear from this work that the model needs some further work tweaking before it can be used to optically dark systems, and therefore becoming a more widely used method.

## Data Availability

The data that support the findings of this study are available from the corresponding author upon reasonable request.
